# Atlanta Academy of Medicine

**Published:** 1874-12

**Authors:** A. W. Calhoun

**Affiliations:** Reporter


### Atlanta Academy of Medicine.

A. W. CALHOUN, M.D., REPORTER.

ATLANTA, GA., October 19, 1874.

Dr. Armstrong, President, presiding.

Dr. W. F. Westmoreland had been called to see a lady forty-odd years old, who was thought to be near the change of life. She had been seriously ill for a month or six weeks, and, when seen by him, her pulse was 125 to 130, and feeble, with hectic symptoms. Upon examination per vagina, found the pelvic organs much swollen, cellulitis, a doughy feel and sensibility upon pressure and upon moving, with occasional vomiting. There was a well developed tumor extending from within the pelvis to mid-way between the pubis and umbilicus.

He concluded it was pelvic cellulitis, and directed a nourishing diet and vascular sedatives, with blisters over the tumor. Called again last week, five weeks from first visit, and satisfied himself that the tumor contained a fœtus, whose movements are well marked and easily felt. He could not examine the uterus high up towards its fundus, on account of the indurated condition of the parts. He is not satisfied whether the womb is impregnated or whether there is an extra uterine pregnancy. He inclined, however, to the latter belief. If it is extra uterine, and that can be decided by sounding the uterus, what shall be done? What course is to be pursued? The cervix uteri is about in the condition it should be when the womb contains a five or six months fœtus.

Dr. Battey remarked, if as far advanced as five or six months, the physiological changes ought to decide whether the fœtus is in the uterus or not. We all know the fact that in multiparous women the walls of the abdomen are thin. In Philadelphia, in a case of ruptured uterus, where gastrotomy was made and a fœtus removed, the walls of the uterus were found to be very thin; such might be the case here. He made a report some years ago to the State Medical Association, of a tumor in the inguinal region, in the case of a man who had been under observation for ten years. There was marked fluctuation, and a small, well defined fœtus seemed to be detected, and every part located, as examined by himself and other medical men. The tumor grew, reaching nearly to his knee, and became so burdensome that it was necessary to support it in a sack. After the lapse of years he removed it, and found it to be nothing more than a simple fatty tumor; no fœtus, nothing but fat. In several instances he had felt the apparent movements of a fœtus in the abdomen where there never was a fœtus.

Dr. W. F. Westmoreland, in reply to the inquiry into the reasons of his faith in the presence of a fœtus, replied that he had felt the movements of the fœtus, and feels satisfied of its presence. Could not hear the fœtal heart, but is strongly of the opinion that a fœtus exists in the tumor.

Dr. Battey thinks it is necessary to clear up the diagnosis. In a patient so long emaciated, a good opportunity is offered of making a conjoined examination. So unpromising are the results of operations for the removal of a fœtus thus located that it is a difficult problem to solve. Nature oftentimes satisfactorily clears it up. He thinks it a good plan to interfere by gastrotomy, and remove the fœtus and leave behind the placenta, allowing the cord to hang out the wound and act as a tent, and thus permit of drainage. He believes it dangerous to leave it to nature. The placenta is left to prevent hemorrhage; it softens and is broken down and discharged through the abdominal opening. More success is achieved in this way than by taking the placenta away, and running the risk of immediate, as well as of secondary, hemorrhage.

Dr. W. F. Westmoreland thought it would be best to remove the placenta and apply styptics to check the internal hemorrhage. He would not hesitate to use styptics in such cases in the abdominal cavity.

Dr. Battey—When we peel the placenta away from the walls of the uterus, we all know of the torrents of blood which often follow. He could see the use of the styptics to the uterus, but how can we go into the abdominal cavity, not knowing the location of the placenta, and depend upon the staying of prodigious hemorrhage with styptics? Such must indeed be a forlorn hope. In ovariotomy, good results are often obtained by establishing free drainage, and allowing the discharge of sanies and septic materials. The history of all operations for extra-uterine fœtation is against the removal of the placenta.

Dr. J. M. Johnson would watch the case closely, and act upon the immediate circumstances. It was impossible to settle definitely the question of operation. The safest plan usually in obstetrics is to leave the matter to nature. If she is unable to meet the demands of the case, then come in with surgery. Nature is very kind; many cases get well under scientific treatment; others get well in spite of bad treatment.

---

Atlanta, November 3, 1874.

Dr. Armstrong, President, presiding.

Dr. J. G. Westmoreland asked how the treatment for dysentery—large doses of ipecac and laudanum—acted?

Dr. Woodhull's opinion is, that ipecac is probably a stimulant to the sympathetic system, and its action in cholera morbus is due to this fact. Acute dysentery is not an inflammation, in the usual sense of the term, but a trouble dependent upon a derangement of this system of nerves.

Dr. W. F. Wertmoreland said that the paper of Dr. Woodhull had changed his opinion of many diseases, as cholera, diarrhœa, dysentery, etc. It has opened up a new channel of thought upon similar diseases, and thinks the profession will appreciate Dr. Woodhull's views.

Dr. J. M. Johnson expressed his pleasure at having heard the reading of the paper—has heard nothing so clear and fair in the deductions. The results are wonderful—would be especially so in civil practice. In the general practice of medicine, such results would not perhaps be attained. The Government supplies good and nourishing food always; the poor, and sometimes the rich, purchase bad food. He does not agree that the disease is always dependent upon derangement of the sympathetic nervous system. He is aware that, when this system is in trouble, these evils oftentimes follow; but, in general practice, the disease is dependent upon imprudence in diet and inattention to the stools. Was struck with the views of the pathology and etiology of the disease. He had so long considered ipecac an emetic that he had been afraid to give it in large doses. Gave it in a case of dysentery in a pregnant woman—3 grs. ipecac and $\frac{1}{8}$ gr. opium, every three hours; forgot to caution her against drinking water, of which she partook freely, and vomited proportionately. Directed her again to continue the remedy, and leave off water; as a result, the stools ceased, pulse became normal, appetite increased, and she was cured. He is thoroughly satisfied it is a great remedy. Two hundred years ago Helvetius imported it from Brazil, and worked wonders with it as a secret remedy. The King of France purchased the secret for an enormous sum, and, besides, conferred distinguished honors upon him. He had noticed that no success had resulted in some hospitals from the use of ipecac. Cream tartar once cured everything that came into the hospitals. Was once traveling with a man who got down every five minutes to evacuate his bowels; cured himself by eating a number of raw turnips. Nature does not regard any of our ideas. Patients get well under all circumstances, even when left alone. We know that quinine will arrest fever, but generally we don't know the effect of remedies. Still, he has great confidence in remedies. The time will come when, instead of giving twenty grains of quinine or its salts, we will give only one-half grain or so, and reach the same result. The same held good in regard to the poppy, when now we have so many salts from it. No man has gone so far into the uses of this remedy as Dr. Woodhull, and he deserves honor for it. Hopes to have it when printed, and intends to put its rules into practice.

Dr. W. F. Westmoreland—I propose to report two cases of the same disease, in which the same remedy was used—in one with the most marked and prompt relief of all the symptoms, while in the other the remedy, applied in the same way, greatly aggravated the suffering of the patient and added greatly to the intensity of the disease. Both patients were suffering with gonorrhœal orchitis, and the remedy used was collodion, and applied in both cases as suggested by the surgeon of *Val de Grace*, the great military hospital of Paris. In 1853 this surgeon, whose name has escaped me, published to the world that he could cure any recent case of gonorrhœal orchitis in a few days. For a time it was the popular remedy in the city, but soon fell into disrepute, and for a time was almost abandoned by the profession.

I propose to illustrate, by the two cases above alluded to, why it is that, in some cases, we have the most marked benefit, with the prompt and permanent arrest of the inflammation, and in other cases all the symptoms greatly aggravated. The first case of the two was a gentleman of middle age, good constitution; the gonorrhœa having existed for several weeks; the orchitis dating only a few days, and evidently on the increase—I mean that the swelling was rapidly increasing. The collodion was applied with a camel's-hair brush, and thoroughly applied, covering the entire scrotum upon the side of the inflamed testicle, and extending half or two-thirds over the scrotum covering the sound testicle. The application was made late in the evening, and my patient was impressed with the importance of remaining quiet until I saw him the next day. He informed me that, in an hour after the application, or before the burning sensation, which is always the result, ceased, that the pain in the testicle and whole scrotum became intense, and increased in intensity until it could no longer be borne. He attempted to remove the collodion covering, but found it almost impossible, as the precaution of removing the hair from the scrotum, before the application, was omitted. He at last, by clipping each hair with a pair of scissors, finally succeeded in removing the collodion, with great relief to his intense suffering. Upon my next visit, he told me, in the most excited manner, that nothing could induce him to pass through another such ordeal—that it was the most intense suffering of his life.

Some months later, I was called to see another case, similar in character. The gonorrhœa and orchitis had existed about the same length of time. In this case, I applied a half dozen leaches the day I saw him, and next day a half dozen more, and then applied the coating of collodion with the most marked and permanent relief—the patient being able, in a few days, to attend to his ordinary duties, when, without the application, he would not have been able to attend to business in three times the length of time.

Why the different results in the two cases? To solve this, it is important to first determine the action of the collodion in orchitis. At first it was supposed that the relief was the result of the direct action of the remedy on the inflamed organ. While in some cases there may result some beneficial influence in this way, yet there is no doubt in my mind that the only influence of any value is the uniform compression of the inflamed organ, and its suspension, which is mnch more perfect if the entire scrotum is covered than can possibly be had with a suspensory bandage.

In my first case, the collodion was applied, or the compression was made, while the inflammation was progressing and the testicle rapidly increasing in size. The collodion compressing the inflamed organ. In the second case, the acute inflammation was arrested before the compression, by means of the collodion, was made.

Dr. Woodhull mentioned the fact that small rubber bands, as used by stationers, were being used as a bandage in this affection.

Dr. Cumming asked if tapping the tunica vaginalis, before applying the bandage, is not efficient? He always does this: secures the testicles, by a band applied above, before applying the bandage.

Dr. Battey called attention to the use of a rubber bag, as an application to the parts; also, the use of a short rubber condom; also, to the treatment by accupuncture, passing the needles deep into the testicle.

Dr. Woodhull showed Esmarch's apparatus for bloodless amputations, and the manner of its application.

---

Atlanta, November 9, 1874.

Dr. Armstrong, President, presiding.

Dr. Taliaferro reported the case of a woman, advanced about six months in pregnancy, who, on the night of the recent illumination, while walking from her house towards the Square, felt a sudden pain in the bowels, and immediately thereafter an immense gush of water poured from the vagina—according to the woman's statement, about two or three gallons. After resting a few moments, she continued her walk, until another, but not quite so free a flow of water, followed. She was taken home, and the doctor gave her a full dose of laudanum per rectum, and left her quiet in a short time. On visiting her the next day, he found several folded quilts, on which she had been placed, saturated with the water which had passed during the night. After remaining quiet for a few days, during which time she suffered no pain, she got up and has been going about since, suffering no inconvenience, although her husband asserts that she passes about two quarts of water from the vagina each day. Movements of the fœtus are very perceptible, and everything getting along well. Asks from what point this water comes?

Dr. J. M. Johnson was called, twenty years ago, to see a patient twenty miles in the country, and found her to be advanced, apparently six months, in pregnancy, but even larger than women usually are at this period. Was sent for again, and found her three times larger than before, but could discover no signs of a fœtus. A few days more found her literally filled with water. Around the neck of the uterus there was fluctuation, and while examining this point the sac ruptured and gallons of water flowed out. She went on to the full period, and a child was born, with the usual amount of water at birth.

Dr. W. F. Westmoreland thinks it not unusual to have discharges from the womb two or three months previous to birth. Takes it to be a cyst within the walls of the fœtal membranes, which bursting, the waters flow out, and allow the process of gestation to progress. Thinks the cases of Doctors Taliaferro and Johnson to be such.

Dr. Armstrong had a patient, in the seventh or eighth month of pregnancy, who had had pains and lost much water. Examined her, supposing labor was beginning. The abdominal walls were loose, but there was no dilatation of the os. He decided she might have had cysts within the walls of the membranes. After several weeks, was called, on account of another discharge of water, and found the same state of things as before. Was again satisfied it was the rupture of a cyst. She was finally delivered at full time. Thinks whenever the real membranes break, labor takes place.

Dr. W. F. Westmoreland had a patient, who had borne three or fóur children, and was supposed to be dropsical by the several physicians who had seen her. Tapping was proposed, but was postponed. She was enormously swollen, and was really in a "terrible condition." He afterwards thought the movements of the fœtus could be felt. The womb was distended to its utmost. The decision was ascites connected with pregnancy, and the trocar was ready to be used. The day before deciding to operate she had some pain, and more on each day following; finally a labor pain and a fœtus, but she still didn't diminish. The membranes being tense, he broke them through with the finger, and with gallons of water came another fœtus, and still she was as large as a woman ordinarily is at full term. Another membrane was ruptured, and another fœtus; six or seven gallons of water, and three babies! She recovered, and went home in a short time.

---

ATLANTA, November 17, 1874.

Dr. Armstrong, President, presiding.

Dr. J. M. Johnson reports the case of milk leg in Mrs. B., who was delivered, October 23, of a three or four pound child. She had morning sickness during entire pregnancy, retaining not one in seventy-five meals, and being, as a consequence, much reduced in flesh. During the last two months, he does not think she retained a single mouthful. It was sometimes held for a day, and there must have been some assimilation, but it was then cast up as it had been eaten. Enquired why such a state should exist? The abdomen was hard and not much enlarged, the child being bound down as with hoops. The fundus of the uterus reached as high as the ensiform cartilage. He made efforts to relieve this by position, but was unsuccessful. It was a long time before the child was born, which was supposed to be dependent, as also the size of the child, upon the tightness of the abdomen and the binding of the uterus. The contractions were regular, but the muscular power was quite weak, and the patient was exhausted when delivered. The discharge for five days was as usual, then became too free. On the eleventh day it ceased, when she complained of soreness about the umbilical region. Upon using the warm douche to restore the lochia, it returned after twelve or fifteen hours. The third day afterwards she had soreness over the entire abdomen, and on the fourteenth day pain in the womb, which was treated with hot water, etc. On the morning of the 15th pain in the hip came on, and in a few minutes the pain in the abdomen and womb subsided. Within two hours there was violent pain in the region of the groin, and, in the course of half an hour, pain and soreness in the calf of the left leg; half an hour longer, pain in the foot, and, in a few hours, swelling from the groin to foot. In the meantime, treatment had been instituted to relieve the pain in the abdomen, and the limb was rubbed upwards from the foot to the body, every two or three hours, as hard as she could bear it. This was continued till the swelling was gone and the cord-like feeling of the veins had disappeared. She was taken on the 15th with pain in the limb, and on the 17th she was entirely relieved, with the exception of the soreness which disappeared on the 19th, and by no other treatment than rubbing the limb and the use of enemas of black drop. The Doctor asked to have the views of the Academy upon the case, as to the treatment—whether the result is merely accidental or due to the mode of treatment? Shortly after the disease made its appearance, there seemed to be occlusion of the veins, and the soreness indicated violent inflammation. It is a rational view to suppose it phlebitis, but his observations did not lead him to this conclusion. He has seen many cases of milk leg, but never one so violent, in all respects, and with so happy results; the swelling and pain were all gone by the third day, and the soreness by the fifth. From the time of the appearance of soreness in the abdomen up to the time of the decline in the swelling, she had a rigor each day, followed by some fever, but this ceased as the swelling disappeared.

Dr. W. F. Westmoreland thinks Dr. Johnson's case is the strongest evidence of milk leg being a nervous disease. Several years ago Dr. Coe, of Atlanta, took the ground that it was not crural phlebitis, as was being then taught in this country and in Europe. This case illustrates Dr. Coe's idea of its nervous character. Dr. C. took the position that it was a reflex nervous action, depending upon nervous irritation in the womb. Meigs first called attention to the fact, that amongst the first symptoms of the disease, contraction of the gastrocnemius muscle and pain in this portion of the limb, were prominent. Dr. Coe asked how could phlebitis produce contraction of this muscle? Milk leg is rarely fatal, phlebitis very fatal; hence the disease cannot be an inflammation of the crural veins, but a nervous trouble. Dr. Johnson's treatment accounts for the rapid cure. Warm applications to the womb, the douche, etc., are of the greatest service.

Dr. J. M. Johnson thinks phlegmasia dolens a mixed disease. There is a large deposition of lymph in the areolar tissue, and the swelling is largely due to this. Preceding this, there must be some clogging of the veins—else how do you account for their hardness? It is not entirely a reflex action from the uterus. Dr. Coe throws out the idea of inflammation in the veins. How do you account, then, for the tenderness in the parts? He agrees as to the fatality of the one and the non-fatality of the other. Phlebitis is rarely cured, yet it would not be safe to say we had

*no* phlebitis in this disease, for we have the symptoms of such an inflammation. In phlegmasia dolens, there is a tendency to abscess, and then the patient recovers. Sometimes death ensues. Is inclined to consider this a mixed form of disease. In dissections, there is nearly always phlebitis, and thinks the disease would invariably go on to phlebitis if let alone. It used to be the rule to do nothing more for the patient than make hot applications and keep the limb warm in blankets. Knows that some deaths did occur at this time, and knows also that in some of these cases phlebitis was present. The best treatment is warm applications to the womb, the douche, etc., and rubbing the limb. He has great confidence in Dr. Coe's ideas; but when we know that the uterine sinuses are open, and the foul substance is passing from the womb into the veins, it is reasonable to suppose that clots should form in the veins as a result. We do not have the redness along the veins, but we have the hardness and other symptoms of phlebitis. But, by re-establishing the uterine flow, this is overcome.

Dr. Cumming said—We have no other symptoms of phlebitis than the swelling of the leg. Has had excellent relief by acting upon the nervous system. Asked if it was not the experience of the Academy that such cases follow a want of nutrition during pregnancy, which brings about serious nervous trouble? Thinks it necessary to keep up nutrition during pregnancy, to prevent such a trouble. Asked if any one had tested the temperature in these cases?

Dr. Taliaferro has doubts as to the disease being inflammatory, but has no well established views as to its pathology.

Dr. Baird said—If this theory of Dr. Coe is true, why is it, in other nervous irritations of the womb, we never have this reflex action?

Dr. Cumming asked what the per-centage was of cases of phlegmasia dolens to the thousand deliveries? No one had given any statistics.

Dr. Holmes agrees with Dr. Johnson that the veins are clogged up by their absorption of morbid matter, forming a thrombosis and producing hardness, swelling, etc. Rubbing the limb upwards relieves this stoppage and re-establishes the circulation. This, with opium, relieved Dr. Johnson's case. The treatment, as practiced by Dr. J., is quite new and very practical.

Dr. J. M. Johnson had a case, eight years ago, just after reading Dr. Coe's paper, and put into practice the same treatment as now. Found the same trouble in accounting for the embolism—how it began, what produced it, and how it got there? This is a difficult problem, but still it is a fact, and he is satisfied that the blood does not pass through the veins. The object in pressing the limb upwards is to relieve the limb of its exudation, and the vein of its obstruction. Wherever swollen legs exist, an irritable womb has existed throughout pregnancy. Had a case where the woman became blind by effusion into the cavity of the orbit; there was irritability of the womb; since then she has had no such trouble—never has seen these troubles without a connection with irritability of the uterus.

Dr. Love asks what becomes of the thrombus? and mentions Dr. Simpson's plan (of Edinburgh); also spoke of a cotton envelope and oil cloth, which retained the perspiration, it acting as a poultice; and mentioned the use of rubber rollers around the cotton.

Dr. Boring had a lady patient of ordinary health, who had had six children. For the last ten years had attacks resembling rheumatism in the feet; also tonsillitis and polypi. Had removed several of the latter. She moved away, and had a severe attack of rheumatism. On the 9th of October she was confined, and got along well. On the fifth day she complained about her feet, the pain occurring particularly at night. Pressure on the inside of the thighs gave pain in the feet. Any stimulating application gave pain; thought it was gout; called in Dr. Taliaferro, who is of the same opinion as Dr. B. Dr. T. is not thoroughly convinced it is gout, but it is nearer this than anything else. She suffered intensely with a burning pain in the foot, and had slight tenderness in dorsal spine.

Dr. J. M. Johnson is just treating such a case. Thinks it is sclerosis; there is tenderness in the spine; used needles over the spine and gave iron and salt baths, and she recovered in about three weeks. Treated it as a disease of the spine.

Dr. W. F. Westmoreland thinks it is a case of hysteria, and electricity is the treatment for it.

Dr. J. G. Westmoreland thought a blister to spine would cure in a few days.

Academy adjourned.

